# Natural Sweetener Stevioside‐Based Dissolving Microneedles Solubilize Minoxidil for the Treatment of Androgenic Alopecia

**DOI:** 10.1002/adhm.202503575

**Published:** 2025-10-07

**Authors:** Junying Zhang, Tianyu Shao, Hailiang Li, Luying Zhu, Lamyaa Albakr, Nial J. Wheate, Lifeng Kang, Chungyong Wu

**Affiliations:** ^1^ Department of TCMs Pharmaceuticals China Pharmaceutical University Nanjing 210009 China; ^2^ School of Pharmacy Faculty of Medicine and Health University of Sydney Pharmacy and Bank Building A15 Sydney New South Wales 2006 Australia; ^3^ Department of Pharmaceutics College of Pharmacy King Saud University Riyadh 11451 Saudi Arabia; ^4^ School of Natural Sciences Faculty of Science and Engineering Macquarie University Sydney New South Wales 2109 Australia; ^5^ Department of Pharmaceutical Analysis China Pharmaceutical University Nanjing 210009 China

**Keywords:** dissolving microneedles, minoxidil, solid dispersion, solubilization, stevioside

## Abstract

Androgenetic alopecia (AGA) is a prevalent form of non‐scarring hair loss. Standard treatments, which include minoxidil (MXD) tincture and foam, face challenges due to MXD's water insolubility and poor skin permeability. The result is extended treatment duration and reduced therapeutic effectiveness. This study utilized stevioside (STV), a natural sweetener derived from the Stevia plant, as a novel solubilizing excipient and microneedle (MN) material. A solid dispersion of STV with insoluble drugs is developed and molded into an MN patch. STV significantly increased MXD's solubility to 47 mg mL^−1^ in water, ≈18 fold higher than the control. STV solubilizes MXD by forming micelles in aqueous solution with a critical micelle concentration of 15 mg mL^−1^. In vitro skin permeation studies showed cumulative drug release of 85% and 18% skin retention for the MN patch, which indicated excellent drug absorption into the skin. Animal studies demonstrated that the MN patch significantly promoted hair growth. There is a significant increase in hair follicle transition to the growth phase, which resulted in 67.5% coverage of the treatment area by day 35. Collectively, the results highlight the potential of the STV MN delivery system for the treatment of AGA.

## Graphical abstract

This study addresses limitations of minoxidil (MXD) in treating androgenetic alopecia by using stevioside (STV) as a solubilizing agent and microneedle (MN) material. STV enhanced MXD's solubility and skin absorption, leading to improved drug delivery and significant hair regrowth in animal models, demonstrating strong potential for AGA treatment.

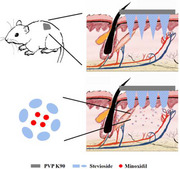

## 1 Introduction

Androgenetic alopecia (AGA) is a common condition marked by progressive hair loss in both men and women. The underlying mechanism involves the conversion of testosterone to dihydrotestosterone (DHT) by 5α‐reductase, which triggers the apoptosis of follicular keratinocytes. This results in increased telogen effluvium, a shortened hair cycle, and the miniaturization of hair follicles.^[^ ^1^, ^2^ ^]^ Beyond its aesthetic effects, AGA can significantly impact the quality of life and the psychological well‐being of patients.^[^ ^3^ ^]^

Commercial treatments for AGA include topical minoxidil (MXD) and oral finasteride. MXD is the only FDA‐approved over‐the‐counter medication for male and female pattern hair loss.^[^ ^4^ ^]^ The efficacy of traditional formulations of MXD is limited due to poor water and skin permeation, thus requiring frequent applications, which complicates adherence. Adding ethanol or propylene glycol excipients improves penetration but can cause unpleasant side effects when MXD crystallizes after solvent evaporation. These side effects include itching, rash, dandruff, and allergic contact dermatitis. As a result, non‐alcohol‐based MXD formulations have been developed to improve patient comfort and adherence.^[^ ^5^ ^]^

Microneedling, an innovative adjunctive treatment for AGA, involves the use of fine needles to create micro‐punctures in the skin. Emerging evidence suggests that microneedling can independently stimulate hair growth by promoting dermal remodeling and enhancing blood flow to hair follicles. This minimally invasive procedure holds promise as a useful therapy for patients with AGA, particularly for those who do not respond to conventional treatments or are unable to use MXD due to its contraindications, such as hypertension.^[^ ^6^ ^]^

Combining microneedling treatment with MXD solutions improves the permeability of the skin and facilitates deeper penetration of MXD into the hair follicles, which both act to enhance efficacy. Clinical studies have shown that this type of combination therapy significantly promotes better hair growth when compared with MXD monotherapy.^[^ ^7^ ^]^ The hair regrowth strategies based on MNs are summarized in **Table** **1**.

**Table 1:** Hair regrowth strategies that utilize MN‐based drug delivery systems.

Materials	Drug(Formulation)	Indication	Dosage (MNs)	Application mechanism	Refs.
roller‐MNs	MXD NPs	AGA	10 mg	The roller‐MNs create microchannels in the skin to enhance drug transdermal efficiency. Paste systems successfully boosted perifollicular vascularization, and activated hair follicle stem cells, thereby inducing notably faster hair regeneration at a lower administration frequency on an AGA mouse model.	[9]
roller‐MNs	5% MXD lotion	AGA	/	A dermaroller of 1.5 mm sized needles was rolled over the affected areas of the scalp in the longitudinal, vertical, and diagonal directions until mild erythema was noted. Minoxidil only was then applied 24 h after the microneedling procedure.	[12]
HA‐MNs	MXD	CA	6 mg	HA provided a better environment for cell‐cell adhesion and cellular functions, such as HDP cell proliferation, migration, and aggregation. It also played a role in controlling hair growth.	[13]
PVA‐MNs	MXD(PLGA‐NPs)	AGA	56.3 ± 9.2 µg	MNs as drug reservoirs with sustained release of MXD‐loaded microspheres for more than 2 weeks.	[11]
HA‐MNs	MXD NLC	AGA	538.5 ± 6.9 µg	MXD NP‐loaded MN system that was able to promote hair follicle transformation from the telogen phase to the anagen phase.	[14]
Gel‐MNs	MXD	AGA	16 mg	Implantable MNs designed to break at the interface between the swollen tip and the non‐inserted column upon the absorption of body fluids.	[15]
PVA‐MNs	MXD(MM‐NPs)	Alopecia	/	MNs containing MXD and MIO (mesoporous iron oxide nanoraspberry) that demonstrated 8‐fold improvement in hair regrowth through a combination of drug treatment and a magnetic heat effect.	[16]
CAMC‐MNs	FIN (Powder)	AGA	209.8 ± 8.7 µg	MNs that demonstrated sustained release of FIN for 3 days, which increased the number and density of hairs.	[17]
CAMC‐MNs	VPA (Powder)	AGA	1M	Up‐regulation of Wnt/β‐catenin and alkaline phosphatase activity in human dermal papilla cells, and thus, induction of HF regeneration.	[18]
HA‐MNs	FIN (NLC)	AGA	47.4 ± 0.9 µg	NLC enhanced HF accumulation of FIN which resulted in FIN inhibition of DHT conversion, upregulation of the signals for β‐catenin, IGF‐1, and VEGF, and downregulation of the signals for SRD5A2, TGF‐β1, and IGF‐1.	[19]
HA‐MNs	Ceria	AGA	8.7 ± 0.8 µg	Scavenged ROS and promote angiogenesis in the perifollicular microenvironment simultaneously to facilitate hair regrowth	[20]

Abbreviations: CA, chemotherapy‐induced alopecia; CAMC, carboxymethylcellulose; FIN, finasteride; Gel, gelatin; HA, hyaluronic acid; HDP, hair dermal papilla; HF, hair follicles; IGF, insulin‐like growth factor; MM‐NPs, minoxidil and mesoporous iron oxide nano‐raspberry nanoparticles; NLC, nanostructured lipid carriers; NP, nanoparticle; PLGA, poly(lactic‐co‐glycolic acid); PVA, polyvinyl alcohol; ROS, reactive oxygen species; SRD5A2, type II 5α‐reductase; TGF‐β1, transforming growth factor‐β1; VEGF, vascular endothelial growth factor; VPA, valproic acid.

Moreover, a meta‐analysis of randomized clinical trials highlighted that MN therapy combined with MXD significantly increased hair count and diameter when compared with topical MXD monotherapy.^[^ ^8^ ^]^ Metallic solid MNs have been studied to enhance the penetration of MXD by creating puncture holes in the surface skin. Li and colleagues have studied the use of metal MN rollers with an MXD paste formulation to improve MXD absorption.^[^ ^9^ ^]^

The use of dissolving MNs loaded with MXD has also been studied. Unlike solid MNs, which may cause skin inflammation and dosing inaccuracies, dissolving MNs offer precision drug delivery, avoid sharps waste products, and can accommodate the delivery of multiple drugs, thus providing a multi‐pathway and multi‐targeted approach to hair regeneration.^[^ ^10^ ^]^ Kim and colleagues have reported MXD‐loaded hyaluronic acid (HA) dissolving MNs for improved hair growth in a chemotherapy‐induced alopecia model.^[^ ^1^ ^]^ The MXD‐loaded MNs enhanced hair dermal papilla cell proliferation through CD44 and Akt signaling.

Another study examined a dissolving MN patch with biodegradable MXD laden microspheres for long‐acting hair regrowth therapy.^[^ ^11^ ^]^ Yin and colleagues developed this patch to enhance bioavailability and reduce administration frequency, thereby improving adherence. The patch delivered MXD‐encapsulated poly(lactic‐co‐glycolic acid) microspheres into the skin, which sustained release over a two‐week period, as well as providing mechanical stimulation. This approach used a lower drug dosage when compared with daily topical application, which offers a safe and effective strategy for long‐term hair regeneration in clinical settings.^[^ ^11^ ^]^

In light of recent progress, we investigated for the first time a new delivery system that utilized stevioside (STV), a natural sweetener, as the material for the production of MNs. STV is minimally metabolized and excreted without accumulation. Toxicology studies have shown that STV is safe and does not exhibit teratogenic, carcinogenic, or mutagenic toxicity.^[^ ^21^, ^22^ ^]^ It was approved by the European Commission as a food additive in 2011, and since then, STV has become widely used in the food industry.^[^ ^23^ ^]^ As a tetradic diterpenoid glycoside, STV is an amphiphilic molecule, and hence, may have solubilization functionality similar to a surfactant.^[^ ^24^ ^]^

We have previously reported that glycosylated compounds, such as mogroside V,^[^ ^25^, ^26^ ^]^ glycosylated hesperidin,^[^ ^27^ ^]^ and glycosylated rutin^[^ ^28^ ^]^ can be used as drug carriers to solubilize insoluble drugs. The carriers can self‐assemble to form micelles in aqueous solutions with typical hydrophobic cavities within which hydrophobic drugs can enter through intermolecular interactions.^[^ ^29^ ^]^ It is believed that STV can self‐assemble into core‐shell aggregates in aqueous solution and carry small drug molecules; no complex preparation is needed as the drug loading process is simplified by spontaneous micelle formation.

Using STV as a novel solubilizing agent, we incorporated MXD into a dissolving MN patch, which was used to enhance the solubility and bioavailability of MXD and overcome the limitations of traditional carrier single‐function approaches. The MN patch simplifies the treatment regimen by providing a more convenient, long‐acting option that ensures controlled and prolonged drug release directly to the targeted area. This method not only enhances drug penetration into skin but also eliminates other issues associated with traditional topical formulations, such as slow onset of action and/or inaccurate dosing. By combining the advantages of microneedling with the therapeutic benefits of MXD, and utilizing STV's unique properties, this approach holds significant potential for improving clinical outcomes in the treatment of AGA.

## 2 Results and Discussion

### 2.1 Solid Dispersion (SDP) Preparation and Characterization

**Figure 1:**
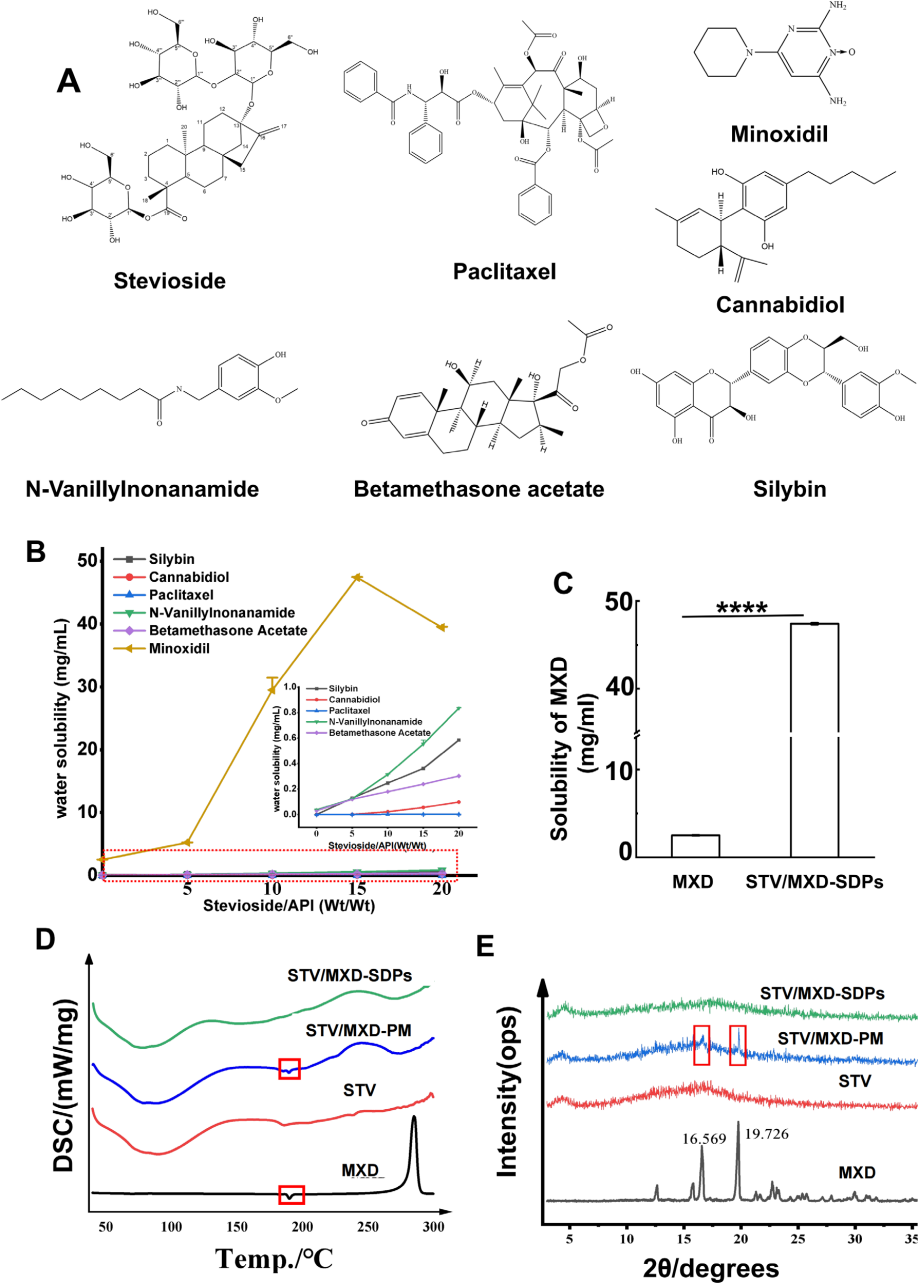
A) The chemical structures of stevioside, paclitaxel, N‐vanillylnonanamide, MXD, cannabidiol, betamethasone acetate, and silybin. B) Apparent solubility of insoluble drugs in solid dispersions. The apparent solubility of the change mass ratio of STV to different insoluble drugs (mass ratio: 5:1, 10:1, 15:1, and 20:1) (n=3). C) Solubility of pure MXD and MXD in STV/MXD‐SDPs with a mass ratio of 1:15 (n=3). D) DSC thermograms and E) PXRD spectra of STV, MXD, STV/MXD‐PM, and STV/MXD‐SDPs. (****p < 0.0001).

To study the solubilization effect of STV, six common insoluble drugs were selected: paclitaxel (PTX), N‐vanillylnonanamide (NVA), cannabidiol (CBD), betamethasone acetate (BA), silybin (SLB), and MXD, as shown in **Figure** **1**A. For each drug, a solid dispersion was prepared in an STV matrix using thin film evaporation. The improved drug solubility was then tested, and monitoring of drug encapsulation within the sugar matrix of STV was undertaken using differential scanning calorimetry (DSC) and powder X‐ray diffraction (PXRD).

#### 2.1.1 Solubilizing Effect of STV on Drugs

The solubility of the drugs increased progressively with increasing STV concentration, as shown in Figure 1B. Notably, the solubility of SLB in the STV/solid dispersion increased ≈308.2, 574.0, 839.8, and 1357.2 fold. For CBD, the solubility increased by 48.7, 946.1, 2409.3, and 4252.7 fold. Although the solubilizing effect on PTX, NVA, and BA was less pronounced, it remained significant, as PTX showed a solubility increase of up to 43.6 fold, NVA up to 22.3 fold, and BA up to 10.1 fold.

In the case of MXD, solubility improved with increasing STV concentration, reaching a peak of 47.4 mg mL^−1^ at a 15:1 STV‐to‐MXD ratio (Figure 1C), which is ≈18.4 fold higher than the solubility of just MXD; however, when the STV‐to‐MXD ratio was increased to 20:1, solubility decreased, likely due to the increased solution volume. Consequently, the 15:1 ratio was chosen for subsequent MN development experiments, as it demonstrated the highest apparent solubility.

#### 2.1.2 Differential Scanning Calorimetry (DSC)

The DSC thermograms are shown in Figure 1D. DSC was utilized to confirm the molecular state of MXD in the solid dispersion. Both the pure MXD drug substance and the STV/MXD physical mixture (STV/MXD‐PM) exhibited a distinct endothermic peak ≈at 190 °C, characteristic of crystalline MXD.^[^ ^29^ ^]^ This indicated that MXD remained in its crystalline form in the physical mixture. However, the absence of this endothermic peak in the STV/MXD solid dispersion (STV/MXD‐SDPs) confirmed that MXD was in an amorphous state within the solid dispersion. The increase in endothermic entropy corresponded with an increase in solubility.

#### 2.1.3 Powder X‐Ray Diffraction (PXRD) Patterns

The PXRD thermograms for the four different formulations are presented in Figure 1E. The crystal diffraction peaks of MXD were very distinct, with two main characteristic peaks at diffraction angles 2θ of 16.6°and 19.7°, along with several other moderate fingerprint peaks, which confirmed their crystal structure. In contrast, the diffraction profile of STV was broad with short diffusion peaks, which indicates that it was amorphous. The diffraction pattern of the STV/MXD physical mixture (STV/MXD‐PM) exhibited weaker characteristic peaks than the MXD crystal structure, and many fingerprint peaks were not apparent. Conversely, the diffraction pattern of the STV/MXD solid dispersion (STV/MXD‐SDPs) showed broad diffusion with no characteristic absorption peaks, which indicated that the STV inhibited the formation of the MXD crystal core and prevented crystallization. This confirmed that MXD was in an amorphous state within the solid dispersion.

### 2.2 Solubility Mechanism Study of STV

STV is a natural diterpenoid glycoside, composed of a hydrophobic terpenoid ring and hydrophilic glucose group units, and rhamnose units on both sides. Studies have shown that STV can enhance the solubility of many hydrophobic drugs.^[^ ^30^, ^31^, ^32^ ^]^ However, the exact mechanism of this enhancement remains unclear. Based on the structural characteristics of STV, it was hypothesized that STV functions similarly to the glycosylated sugar molecules reported earlier.

To explore the ability of STV to solubilise MXD, both pure STV and STV/MXD‐SDP were examined using ^1^H Nuclear Magnetic Resonance (NMR) and ^1^H‐^1^H rotating frame Overhauser enhancement spectroscopy (ROESY) experiments. ROESY is a technique related to nuclear Overhauser effect spectroscopy (NOESY) that is used for the detection of nuclear Overhauser effect (NOEs). It is used to analyze molecular structures and can provide information about a molecule's 3D spatial structure, as well as possible chemical reactions and interactions.

**Figure 2:**
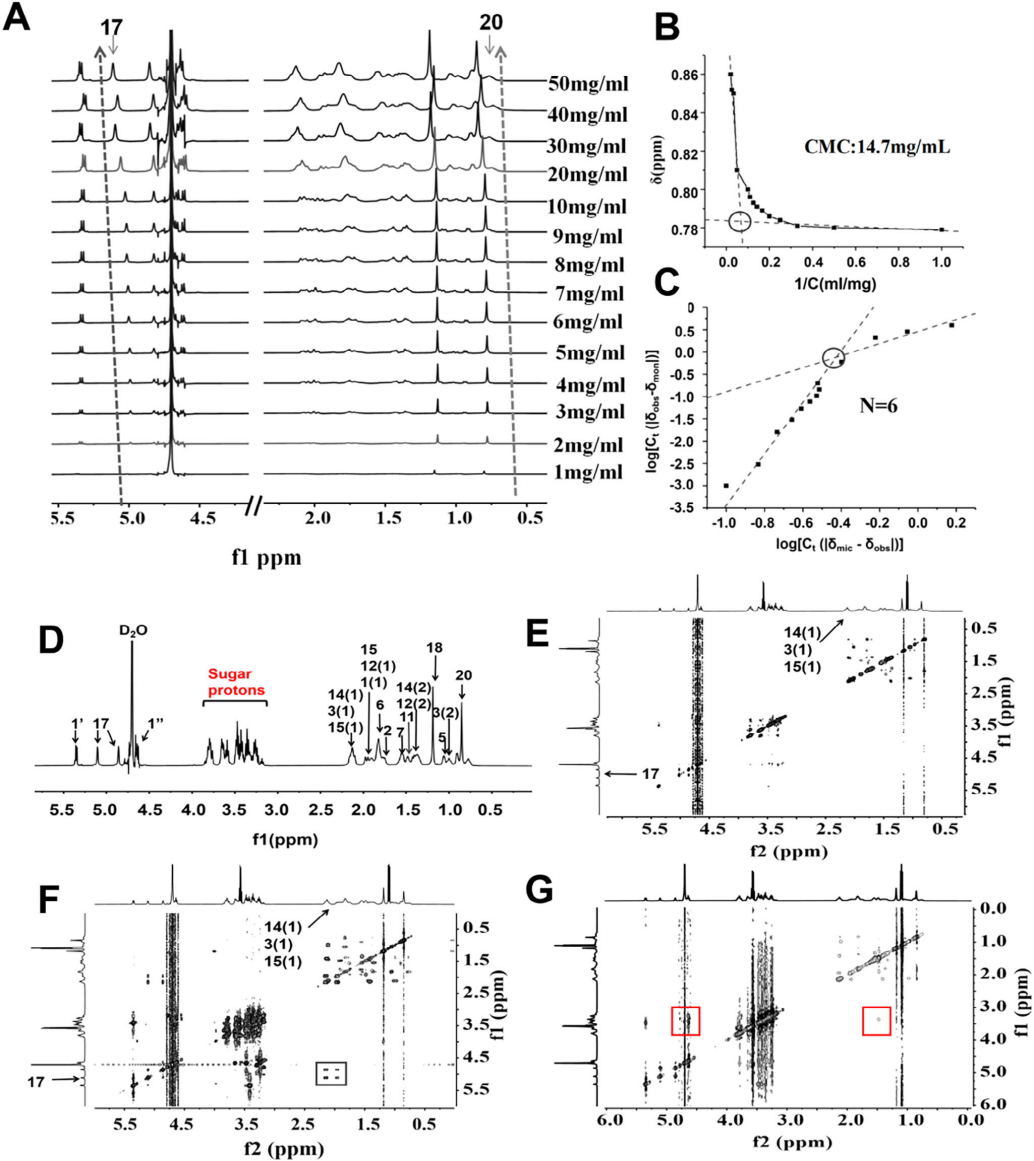
A) The ^1^H NMR spectra of STV at concentrations of 1–50 mg mL^−1^ in D_2_O (δ: 4.0‐5.5, 0.5‐2.5 ppm). B) A plot of δ_obs_ against 1/C for the H‐20 peak from the ^1^H NMR spectra; the intersection of the fitted lines is the CMC. C) A plot of log[C_t_(|δ_obs_‐δ_mon_|)] against log[C_t_(|δ_mic_ ‐ δ_obs_|)] for the H‐20 peak from the ^1^H NMR spectra; log[C_t_(|δ_obs_‐δ_mon_|)] is linearly fitted to log[C_t_ (|δ_mic_ ‐ δ_obs_|)] to obtain two straight lines. D) The ^1^H NMR assignments of STV. E) The 2D ^1^H‐^1^H ROESY spectra of STV at 2 mg mL^−1^ and F) 30 mg mL^−1^ (without MXD). (G) The ^1^H‐^1^H ROESY spectrum of STV/MXD‐SDP at 32 mg mL^−1^ (with MXD). The red box indicates the newly emerged related peaks.

Chemical shift is a crucial parameter in NMR, as changes in the chemical microenvironment within and between molecules can induce changes in the chemical shift of select proton resonances. By monitoring these changes, information about the interactions between the molecules can be gathered. **Figure** **2**A shows that as the concentration of STV increased, there were changes in the chemical shift of its proton resonances. The hydrophilic sugar protons exhibited shifts to both high and low fields, indicating complex aggregation behavior. The terpenoid ring protons, such as the double bond proton (H‐17) and protons of the methyl group (H‐20), shifted to a lower field (a shielding effect), which suggested micelle formation with the terpenoid ring in the center.

To find the critical micelle concentration (CMC), the changes in the chemical shift of the STV H‐20 proton were plotted against 1/C and it produced a CMC of 14.7 mg mL^−1^, as shown in Figure 2B. To find the aggregation number N_agg_, the log[C_t_ (|δ_obs_‐ δ_mon_|)] was plotted against log[C_t_(|δ_mic_ ‐ δ_obs_|)], and the slope of the fitted line was used to deduce the N_agg,_ according to Equation (6). As shown in Figure 2C, two lines with different slopes could be fitted according to the chemical shift value change of STV. The two slopes indicate that the range of concentrations of STV covers the CMC. When the STV concentrations were lower than CMC, the N_1_ of STV was ≈1, indicating that the STV molecules were present predominantly as monomers. But when the STV concentrations were higher than the CMC, the N_2_ was ≈6, which indicated that the STV molecules were aggregating as a hexamer.

When a proton is saturated or inverted, spatially close protons may experience an intensity enhancement, which is termed an NOE. The intensity and location of correlated NOE cross peaks in a ^1^H‐^1^H ROESY spectrum can reflect the strength and arrangement of intermolecular interactions of aggregates and thus provide information on the spatial structure of the aggregates.

Figure 2D shows the ^1^H NMR assignment of STV. There was no signal correlation peak at 2 mg mL^−1^ of STV in Figure 2E. Correspondingly, as can be seen in Figure 2F, for STV at 30 mg mL^−1^, δ 5.36 (1H, s, H1') was correlated with the sugar matrix element at 3.30 ppm, the sugar matrix element at 3.15–3.83 ppm, and the terpene ring protons 0.8–2.2 ppm. Extensive NOEs are observed between the terpene cyclic protons, the glycosyl protons, and between the cyclic and glycosyl protons. This indicated that intermolecular aggregation of STV occurs at high concentrations (Figure 2F). When MXD is encapsulated within STV micelles, the intermolecular distance increases (Figure 2G), which results in weakened STV molecular interactions as evidenced by smaller NOESY cross peaks. In the ROESY spectrum, there were multiple NOEs between MXD and STV. This is consistent with the proposed encapsulation model, where MXD was wrapped by six STV molecules in the hydrophobic cavity.

In the future, the research group will further analyze the molecular features of STV aggregation and drug encapsulation through computer‐aided drug design theory and NMR, to identify other glycosides with solubilization potential.

### 2.3 Preparation and Characterization of the SDP MN

The microneedles were examined under a microscope and showed no visible tip damage, with the MN patches that appeared well‐formed. Figure S1 (Supporting Information) shows that stevioside can effectively form microneedles within a concentration range of 200 to 3000 mg mL^−1^, making it suitable as a tip material.

**Figure 3:**
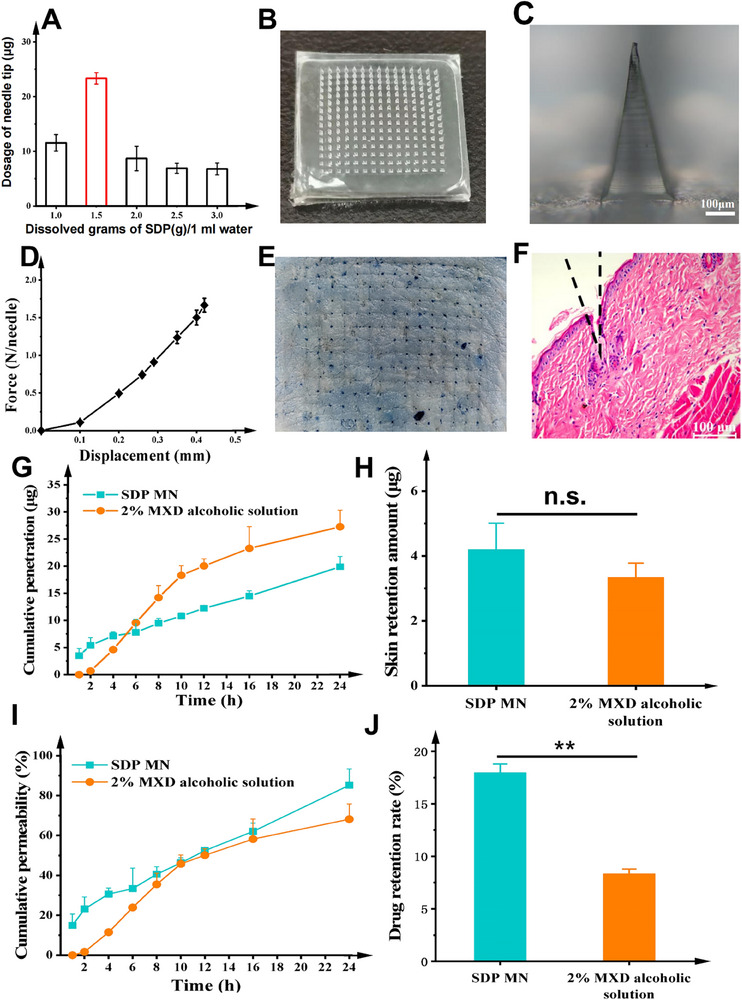
A) The drug loading of the MN tips (n=3) as a function of SDP concentration. B) A photographic image of an SDP MN patch. C) A microscope image of a single needle (Scale bar: 300 µm). D) The force (N)‐displacement (mm) map of SDP MN (n=3). E) Trypan blue‐staining and F) H&E‐staining images of porcine ear skin after MN application (Scale bar:100 µm). G,I) The cumulative penetration curves of the SDP MN and the MXD‐alcoholic solution (n=3). H,J) MXD accumulation in the porcine skin of SDP MN and MXD‐alcoholic solution (n=3).

To increase the drug loading of the needle tip, different concentrations of the solid dispersion were analyzed: 1.0, 1.5, 2, 2.5, and 3.0 g dissolved in 1 mL of water. The MN array was then created using these solutions as the needle tip solution. As shown in **Figure** **3**A, the highest dose that could be achieved was with a tip solution concentration of 1.5 g mL^−1^, which corresponded to a mass of ≈23.3 µg. Morphological analysis of the SDP MN revealed an overall good appearance, featuring a smooth surface and sharp tips (Figure 3B,C). The dimensions of the MNs were: 592.5 ± 4.1 µm for the needle length, 291.9 ± 3.7 µm for the bottom edge length, and 789.5 ± 5.8 µm for the tip‐to‐tip distance.

An MN can penetrate the skin with just 0.1 N of vertical pressure applied to the tip of the needle.^[^ ^33^, ^34^ ^]^ In this study, the MNs showed no obvious inflection point in the pressure during pressure testing, which indicated that no fractures occurred. The maximum force reached was 1.66 N/needle at the end of the test, which met the requirements for being able to pierce the skin (Figure 3D). After the MNs penetrated the skin, it was observed that microchannels had formed within the skin tissue, with a penetration rate of more than 94% (Figure 3E). The microchannels that were created by the MNs, and which could be seen in stained sections of skin, were found to reach below the epidermal layer (Figure 3F). Collectively, the results indicated that the SDP MNs had sufficient mechanical strength for drug delivery applications into skin.

### 2.4 Drug Permeation Studies of SDP MN In Vitro

In the SDP MN (Figure 3G,I) group, rapid (2 h) drug release was observed, as the MN was able to penetrate the stratum corneum and deliver the drug directly to the epidermis and dermis. After the initial fast onsite of drug release, the drug release rate slowed slightly between 2 and 6 h, and reached a steady state after that time period. Cumulative drug permeation reached 85.2% at 24 h, which indicated highly efficient transdermal delivery. In contrast, the 2% MXD alcoholic solution was released slowly over a period of 2 h, and the release rate increased after 2 h. However, the rate of release gradually slowed between 10 and 24 h, and the cumulative permeability at 24 h was only 68.1%, which was significantly lower than that of SDP MN.

The skin retention of MXD (Figure 3H,J) in the SDP MN group and the 2% MXD alcoholic group was 18.0 and 8.4%, respectively. The MXD skin retention quantities were 4.2 ± 0.8 µg and 3.3 ± 0.4 µg, respectively. Although the drug loading in SDP MN was less than half that of the 2% MXD alcoholic solution, the drug retention inside the skin for the SPN MN group was higher than that of the 2% MXD alcoholic group. This result indicated that SDP MN is a more suitable formulation for the treatment of AGA than the traditional commercially available preparation (2% MXD alcoholic solution) and has good application prospects as a topical formulation.

At the late stage of the skin permeation experiments, the drug permeation rate of the 2% MXD alcoholic solution slowed down when compared with the MN group. This may have been caused by the continuous evaporation of ethanol from the skin surface, leading to the accumulation of MXD on the outside of the skin; ethanol acts as both a solvent and skin penetration enhancer. On the other hand, even though the total mass of drug that permeated into the skin in the 2% MXD alcoholic group was higher than that of the MN group, the amount of drug retained in the skin was very limited. However, it is the quantity of drug that is retained inside the skin that matters most, as the drug target (i.e., the hair follicles) resides inside the skin. Therefore, it can be expected that the SDP MN patch had better efficacy than the 2% MXD alcoholic solution. To verify this, animal studies to investigate hair regeneration in mice were undertaken to compare the SDP MN patch against the 2% MXD alcoholic solution.

### 2.5 Animal Studies with the MN Patch

2.5.1

**Figure 4:**
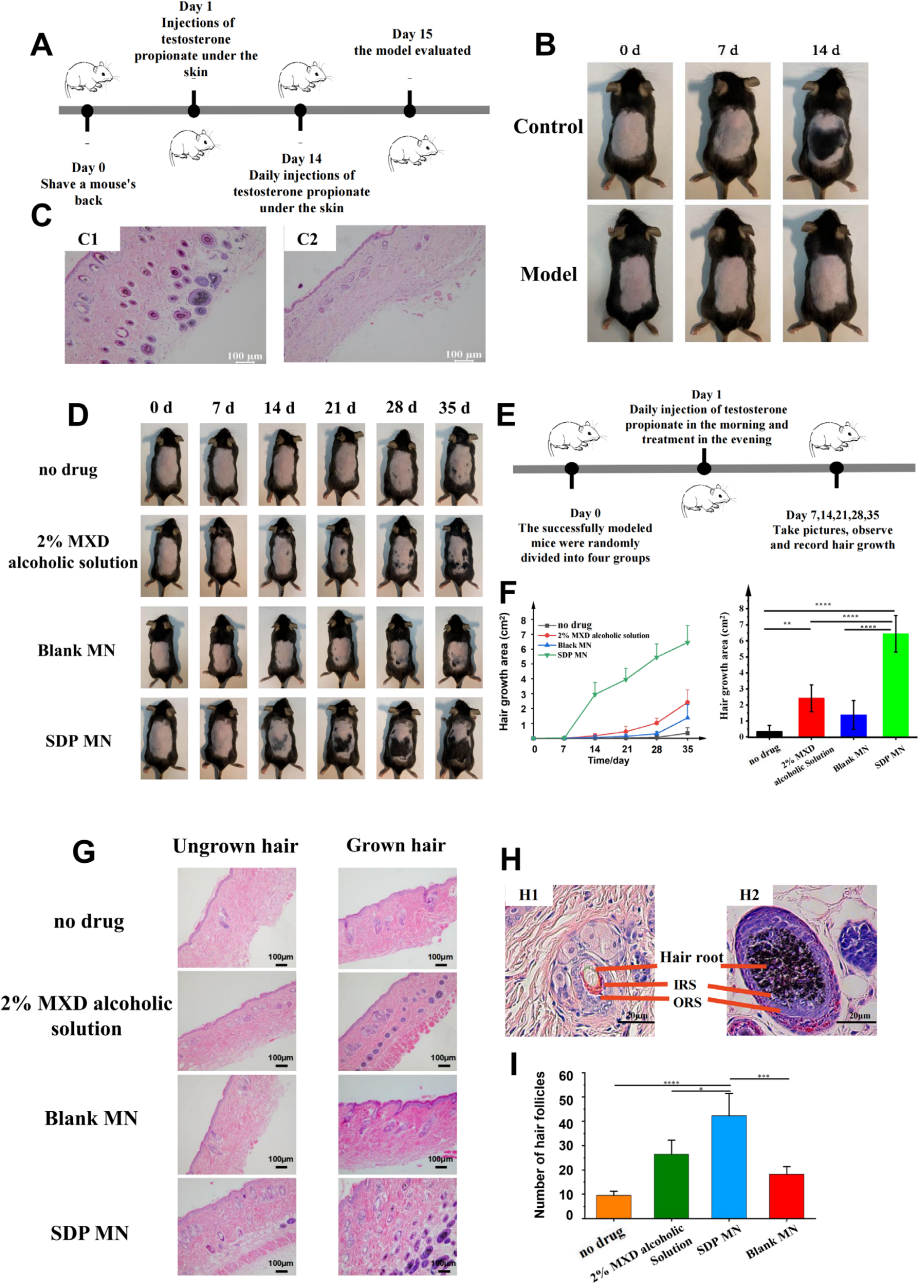
The mice AGA model. A) Schematic representation of androgenetic alopecia modeling. B) Hair growth of mice on days 0, 7, and 14 after modeling. C) Stained sections of the skin from normal (C1) and model mice (C2). (Scale bar: 100 µm). In vivo pharmacodynamic evaluation of SDP MN. D) Hair growth on the back of the C57BL/6 mice in each group during administration (groups as follows: blank MN, 2% MXD alcoholic solution, SDP MN, and no drug). E) A schematic representation of the pharmacodynamic experimental design. F) Left: Hair growth area of each group at days 0, 7, 14, 21, 28, and 35 (n = 6). Right: The hair growth area in each group at day 35. G) H&E staining images of dorsal skin in C57BL/6 mice, left represents ungrown hair parts of mice, right represents grown hair parts of the mice (no drug, 2% MXD alcoholic solution, blank MN, and SDP MN; Scale bar: 100 µm). H) H&E staining images of hair rest period (H1) and growth periods (H2) (ORS: outer root sheath, IRS: inner root sheath. Scale bar: 20 µm). I) The number of new hair follicles in each group (n = 4). (^*^ *p* < 0.05, ^**^ *p* < 0.01, ^***^ *p* < 0.001, ^****^ *p* < 0.0001).

To build an alopecia animal model to test the formulations, testosterone propionate was injected under the skin of C57BL/6 mice, and the subsequent hair loss was monitored over a period of 2 weeks (**Figure** **4**A). The hair growth of the mice during the modeling period is illustrated in Figure 4B. The hair growth cycle is divided into three phases: growth, rest, and regression. On the 7th day, the back skin of normal C57BL/6 mice appeared cyan in color, which indicated the initiation of hair growth. By the 14th day, a substantial area of new hair had developed. In contrast, mice in the modeling group exhibited no new hair growth, with their back skin remaining pink and white. Also, dandruff‐like sebum overflow was observed in the modeling group, which aligned with the pathological features of AGA.

Skin sections from mice not injected with testosterone propionate (Figure 4C1) displayed deep hair follicles located in the lower dermis, as characterized by a large hair papilla and numerous hair follicles. In contrast, mice injected with testosterone propionate (Figure 4C2) showed either an absence of visible hair follicles or sparse follicles with shallow pigmentation and/or incomplete follicular structures. Normal skin slices demonstrated hair follicles that were distributed across the dermis and upper dermis. These observations align with the pathological features of AGA. The subcutaneous injection of testosterone inhibited the anagen phase of hair follicles, which effectively established a histopathologically validated mouse model of AGA.

The results of hair development are shown in Figure 4D, while the course of drug administration is shown in Figure 4E. The mice in the no drug group exhibited minimal hair growth by the 35th day, which indicated that the testosterone propionate had a consistent inhibitory effect on hair follicle growth (Figure 4D). Mice in the blank‐MN group showed a few black spots by the 21st day and green‐gray newborn hair by the 35th day. This was likely the result of MN stimulation that increased the blood supply around the hair follicle.^[^ ^35^ ^]^ In the group of mice treated with the 2% MXD alcoholic solution, small bluish‐black patches started to appear on the skin by the 14th day. New hair growth was slow, but by the 35th day, it covered a portion of the dorsal area. On the other hand, in the SDP‐MN group, a large area of new hair growth appeared by the 14th day. The area of new hair increased rapidly, covering a large area of the mouse's dorsum by the 35th day.

The sizes of the area of the new hair growth for each group are shown in Figure 4F. For the SDP‐MN group, large areas of newborn hair were evident by around the 14th day, after which it maintained a considerable growth rate. By the 35th day, the area of hair growth and the growth rate reached 6.4 ± 1.1 cm^2^ and (67.5 ± 8.1%). The group treated with the 2% MXD alcoholic solution showed significant hair growth around the 21st day; however, there was only a gradual increase in the area of new hair and the hair growth rate, with only 2.2 ± 0.8 cm^2^ and 25.7 ± 6.6% hair growth at the end of the 35th day.

Although the skin retention of MXD was not significantly different between the SDP MN and the MXD alcoholic solution (Figure 3H), the results in Figure 4D showed striking differences between the two groups. This discrepancy may be ascribed to STV's vasodilatory properties, which can enhance blood circulation to the scalp and thereby potentiate the therapeutic efficacy of the MXD‐loaded MN.

The hair follicles in the section of the skin with no newly generated hair are shown in Figure 4G (left). Each group was analyzed under the microscope in four randomly selected fields. The hair follicles in the no drug and blank groups were scattered in the skin with very light pigments, which indicated that they were still resting, as shown in Figure 4H1. The number of hair follicles in the 2% MXD alcoholic solution group increased significantly, but the hair follicles were still only distributed in the dermis, and the growth rate was slow. The number of hair follicles in the SDP MN group was large, distributed in the dermis, and the hair follicles in the deep layer were darker, which indicated that SDP MN was able to promote the hair follicles to transition from the resting phase to the growth phase Figure 4H2.

The newly generated hair of mice is shown in Figure 4G (right). In the no drug group, similar to the blank‐MN group, the hair follicles were sporadically distributed in the upper dermis with light pigmentation. In contrast, the SDP MN group exhibited larger and darker hair follicles with significant melanin deposits, which were concentrated in the center and with a widespread distribution across the dermis.

The results of statistical analyses (Tukey's post hoc test) are shown in Figure 4I. The SDP‐MN group was found to be significantly different from the other treatment groups, but there was no significant difference between the blank‐MN group and the no drug group (*p* > 0.05). This suggested that the observed effects in the SDP MN group were not attributable to the effect of MN or STV alone.

Overall, the results demonstrated that SDP MN can effectively promote hair follicles to enter the growth phase and that the drug delivery system had a significant anti‐AGA effect. The benefits and drawbacks of various technical methods, such as metal or soluble microneedles and sustained‐release systems, should be evaluated in conjunction with specific clinical scenarios. The AGA mouse model used in this study reflected the temporary inhibitory impact of androgens on hair follicles. When compared with similar studies, the results of this study, which examined the combination of soluble microneedles with the long‐lasting effect of sustained‐release patches, represent cutting‐edge development. However, AGA is a polygenic disorder, with its pathogenesis also influenced by lifestyle and other factors. Additionally, the hair follicle growth cycle in humans differs from that of mice. Therefore, further in‐depth and comprehensive studies are needed to evaluate the efficacy of SDP MN on humans.

## 3 Conclusion

Current clinical treatments for AGA are limited, with the commonly used MXD tincture limited by its delayed onset of action, poor infiltration properties, skin irritation, and limited therapeutic efficacy. This study demonstrates the potential of STV as a solubilizing agent and highlights its clinical utility as a novel material in the production of MNs. The MN patches enabled minimally invasive penetration of the skin, and thus, they were able to deliver MXD directly into skin tissue and rapidly induce hair follicles into the growth phase with melanin aggregation. This dissolving MN patch formulation offers an innovative transdermal approach to the treatment of AGA.

## 4 Experimental Section

### Materials

STV (purity 95%) was purchased from Shanghai Bidd Pharmaceutical Technology Co., Ltd. Paclitaxel (PTX, purity 99%), *N*‐Vanillylnonanamide (NVA, purity 95%), MXD, cannabidiol (CBD, purity 97%), betamethasone acetate (BA), silybin (SLB, purity 98%) and PVP K90 were purchased from Shanghai Aladdin Bio‐Chem Technology Ltd. Anhydrous ethanol (analytical purity) was purchased from Shanghai Titan Technology Co., Ltd. Trypan blue was purchased from Sinopharm Group Chemical Reagent Co., Ltd. Polydimethylsiloxane (PDMS) was purchased from Dow Corning (China) Investment Co. Ltd. Testosterone propionate injection (25 mg mL^−1^) was purchased from Hangzhou Animal Medicine Factory (China).

### Solid Dispersion Preparation (STV/Drug‐SDP)

Approximately 50 mg of the water insoluble drugs PTX, NVA, MXD, CBD, BA, and SLB were weighed. Then, the STV and insoluble drug were co‐dissolved in anhydrous ethanol using a 5:1, 10:1, 15:1, or 20:1 mass ratio, and sonicated using a KQ5200DB type CNC ultrasonicator from Kunshan Ultrasonic Instrument Co., Ltd. The solution was evaporated at 50 °C over a period of 1 h using a vacuum rotary evaporator to form a thin film. The film was then removed with a scraper, collected in an EP tube, sealed with sealing film, and refrigerated at 4 °C until use. The solubility of the encapsulated drug was determined using high‐performance liquid chromatography (HPLC, LC‐20AT, Shimadzu, Japan).

### Differential Scanning Calorimetry (DSC)

The DSC experiments were undertaken with a Netzsch DSC 3500 calorimeter (Netzsch, Germany). Sample powders were placed in standard aluminum pans, with dry nitrogen used as the purge gas. An empty crucible was used as a reference. DSC traces were obtained by heating the samples from 40 to 300 °C at a rate of 10 °C min^−1^. DSC traces were obtained separately for MXD, STV, STV/MXD‐physical mixture (STV/MXD‐PM), and STV/MXD‐SDPs.

### Powder X‐Ray Diffraction (PXRD)

Powder X‐ray diffraction spectra were obtained with a Bruker D8 Advance X‐ray diffractometer (Bruker, USA) using a scan speed of 4 ° min^−1^ for a θ range of 3–40°. The Cu Kα radiation source was operated at 40 kV and 30 mA. PXRD spectra were analyzed separately for MXD, STV, STV/MXD‐PM, and STV/MXD‐SDPs.

### Nuclear Magnetic Resonance (NMR)

^1^H NMR spectra were obtained using a Bruker AVIII‐500 nuclear magnetic spectrometer, operating at a proton resonance frequency of 500 MHz. STV powder, weighing 1.000 g, was placed in a microcentrifuge tube, and 1.000 mL of D_2_O was added. The solution was sonicated to create a high concentration mother liquor, which was then used to prepare sample solutions at various concentrations (1, 2, 3, 4, 5, 6, 7, 8, 9, 10, 20, 30, 40, 50 mg mL^−1^). All the samples were analyzed using ^1^H NMR. Specifically, sample solutions of STV at concentrations of 2 and 30 mg mL^−1^, as well as STV/MXD‐SDPs at a concentration of 32 mg mL^−1^, were tested using ^1^H‐^1^H ROESY.

As the concentration of STV passes the critical micelle concentration (CMC), the hydrophobic sections of the molecule aggregate, forming micelles with a hydrophobic core. Changes in the microenvironment of STV can lead to changes in the chemical shift of its protons. As such, the number of STV molecules needed to form a micelle can be determined by monitoring proton chemical shift changes in the NMR spectra. According to the law of mass action, the observed chemical shift value δ_obs_ is shown in Equation (1):

(1) $$\delta_{\mathrm{obs}}=\delta_{\mathrm{mon}}C_{\mathrm{mon}}\;/\;C+\delta_{\mathrm{mic}}C_{\mathrm{mic}}/C$$

where the concentration of the solution does not reach the CMC, the surfactant molecules in the solution exist as monomers:

(2) $$C=C_{\mathrm{mon}}$$

When the concentration of the solution is greater than the CMC, aggregation begins to occur, and there are both monomers and aggregates in the solution:

(3) $$C=C_{\mathrm{mon}}+C_{\mathrm{mic}}$$

(4) $$C_{\mathrm{mic}}=C-C_{\mathrm{mon}}=C-\mathrm{CMC}$$

Substituting Equation (4) into Equation (1), the following can be derived as Equation (5):

(5) $$\delta_{\mathrm{obs}}=\delta_{\mathrm{mic}}-\mathrm{CMC}\;/C\left(\delta_{\mathrm{mic}}-\delta_{\mathrm{mon}}\right)$$

To determine the CMC, two lines can be obtained from plots of δ_obs_ as a function of 1/C where the intersection of the two lines shows the CMC value. The N_agg_ can also be determined based on the chemical shift of the proton resonances as they vary with STV concentration, as shown in Equation (6) and supporting information (SI2):

(6) $$\begin{array}{ccc} \log\;\left[C_{t}\left(\delta_{\mathrm{obs}}-\delta_{\mathrm{mon}}\right)\right] & = & \mathrm{nlog}\left[C_{t}\left(\delta_{\mathrm{mic}}-\delta_{\mathrm{obs}}\right)\right]+\log\left(\mathrm{nK}\right)\\ & & +\left(1-n\right)\times \log\left(\delta_{\mathrm{mic}}-\delta_{\mathrm{mon}}\right) \end{array}$$

In Equation (6), δ_obs_ = observed chemical shift value; C_mon_ = monomer concentration; C_mic_ = total concentration of monomers in the micelles; C_t_ = total concentration; δ_mon_ = monomer chemical shift; δ_mic_ = chemical shift of the aggregate; n is the aggregation number (N_agg_); K is the equilibrium constant for micelle formation.

### STV/MXD Solid Dispersion Microneedle (SDP MN) Preparation

To evaluate the performance of STV as a novel MN material, MNs were fabricated using STV tip solutions within a specific concentration range. Samples of STV/MXD‐SDPs (1.0, 1.5, 2, 2.5, and 3.0 g) were dissolved in 1 mL of water. The MN array was prepared by centrifugation using the STV/MXD‐SDPs as the needle tip solutions with the preparation that was previously found to have the highest drug content in the tip, and a backing solution of 30% PVP K90. A PDMS mold was filled with the tip solution and centrifuged at 4500 rpm for 10 min. Then the backing solution was added, and the mixture was centrifuged at 4500 rpm for 3 min. Finally, the MNs were dried in an oven at 50 °C for 3 h.

### SDP MN Characterization

The morphology of the SDP MNs was examined using a Leica DM6B upright microscope (Switzerland). The mechanical strength of the MNs was assessed using an electric pressure testing machine (ZQ 996A, China). Three individual samples were examined, and the average pressure under the same deformation was calculated. A pressure‐shape variable curve was then plotted, with the shape variable plotted on the x‐axis and the average force of each needle as the y‐axis.

The structure and thickness of pig ear skin (obtained from Shanghai Zimei Technology Co., Ltd.), were very similar to human skin. Frozen pig ear skin was cut to an appropriate size, thawed at room temperature, soaked in phosphate buffered saline (PBS) for 20 min, and then dried with absorbent paper. Next, an MN patch was placed onto the skin and pressed vertically with a thumb for 5 min to insert the tips into the skin. After removing the MN patch, a few drops of trypan blue were immediately applied evenly onto the skin. After 10 min, excess dye was washed off using PBS before photographs were taken to calculate the penetration ratio.

A piece of pig ear skin was taken after applying an MN patch for 5 min to observe the insertion of the micro acupuncture into the skin. The pig ear skin was immediately removed and fixed in a centrifuge tube containing a 4% paraformaldehyde fixing solution for 3 days. The fixed skin tissues were dehydrated, embedded, sliced, dewaxed, and dyed to create hematoxylin and eosin (H&E) slices of the skin before microscope images were obtained.

### In Vitro Drug Permeation Through Skin

In vitro penetration was assayed using a Transthilometer diffusion pool (TK‐12D, China). A 30% (v/v) ethanol‐PBS solution was chosen as the receiving medium for the transdermal release experiment. SDP MN samples were vertically patched onto the skin and pressed for 5 min to facilitate skin penetration. Next, a 2% MXD alcoholic solution (2 µL, ≈40 µg of MXD) was applied to the skin. Then, 300 µL of receiver fluid was collected at 1, 2, 4, 6, 8, 10, 12, 16, and 24 h, and the same volume of fresh fluid was added. HPLC analysis (column: Hedera ODS‐2 C18; mobile phase: methanol and 0.1% acetic acid water (40:60); flow rate: 1 mL min^−1^; detection wavelength: 280 nm) was performed to determine MXD concentrations. Following the in vitro transdermal experiment, drug residues in the skin were determined through chromatographic analysis. The cumulative transdermal permeability was calculated using the following formulae:

(7) $$Q_{n}=C_{n}\times V+\sum_{i=1}^{n-1}C_{i}\times V_{i}$$

(8) $$q_{n}\%=Q_{n}/m\times 100\%$$

where, Q_n_ is the cumulative permeability of the n^th^ sampling point; C_n_ is the measured concentration at the n^th^ sampling point; V is the receiving liquid product in the receiving tank (V = 8 mL); C_i_ is the measured concentration at the i^th^ sampling point; V_i_ is the sampling volume of the i^th^ sampling point (V_i_ = 300 µL); q_n_% is the cumulative permeability of the n^th^ sampling point; and m is the mass of drug in the preparation.

### Hair Growth on Mice After MN Treatment

Male C57BL/6 mice (8–9 weeks) were acquired from Spiff (Suzhou) Biotechnology Co., Ltd, and given 3 days to acclimate. All animal experiment operations were approved by the Institutional Animal Care and Use Committee at China Pharmaceutical University (Nanjing, China) under the ethics approval (No. 2023‐03‐015). All procedures were carried out based on China Pharmaceutical University regulations related to the welfare of experimental animals. Randomization was performed to create 4 groups (6 animals per group): SDP MN, 2% MXD alcoholic, blank MN (microneedles containing only the matrix material STV without MXD), and a no drug group. Before pharmacodynamic experimenting, the AGA mouse model was established. The mice were anesthetized, and then an area of 12 cm^2^ (3 × 4 cm) was shaved on the dorsal skin of the mice using animal scissors and hair‐removing cream from Guangzhou Ruixin Biotechnology Co., Ltd (China). After shaving, the mice were placed back into the mouse cage. The mice in the study were given 5 mg kg^−1^ of testosterone propionate each day. The testosterone propionate was diluted in injection grade soybean oil to a concentration of 2.5 mg mL^−1^. The mice received the testosterone propionate solution each day for 14 days to create the AGA mouse model. Photos of the model mice and the control mice were taken on days 0, 7, and 14, and the model was evaluated after the 14‐day period.

A pharmacodynamic study was conducted on mice with successful back skin modeling. In each group, the mice continued to receive subcutaneous injections of testosterone propionate every morning, and the drug was administered every night. Each mouse in the blank MN and the SDP MN groups was given a corresponding MN patch daily, and the 2% MXD alcoholic (167 µL) group was sprayed once a day. From day 0, weekly photos were taken to record the growth of hair. The area of new hair on each mouse was calculated using Image J, and the area of new hair and hair growth rate of each group were plotted. After 35 days, the mice were euthanized, and the skin with the new hair area was analyzed using H&E staining to determine hair follicle density and anagen/telogen ratios; a parameter widely recognized as the gold standard for assessing hair regeneration in preclinical studies.

### Data Treatment

All experiments were performed at a minimum of triplicate. The data were analyzed using OriginPro 2019b software and SPSS IBM Statistics Viewer. The comparison of data between groups was conducted using a two‐tailed independent *t*‐test, and the comparison of data among multiple groups was statistically analyzed using one‐way analysis of variance (ANOVA). Then Tukey's method was used to determine if there were significant differences among the groups. A *p* < 0.05 value was used to indicate a significant difference. P values were reported as *p* < 0.05, *p* < 0.01, *p* < 0.001, or not significant (ns). The experimental data are expressed as mean±SD.

## Conflict of Interest

The authors declare no conflict of interest.

## Author Contributions

J.Z. performed conceptualization, methodology, investigation, software, validation, visualization, formal analysis, and wrote the final manuscript. T.S. performed methodology, investigation, and software, and wrote the final manuscript. H.L. performed the investigation, wrote the final manuscript. L.Z. performed validation. L.A. performed visualization, wrote, reviewed, and edited the final manuscript. N.W. wrote, reviewed, and edited the final manuscript. L.K. conceptualization, methodology, software, formal analysis, data curation, supervision, project administration, wrote, reviewed, and edited the final manuscript. C.W. performed conceptualization, methodology, software, validation, investigation, data curation, supervision, and project administration.

## Supporting information



Supporting Information

## Data Availability

The data that support the findings of this study are available from the corresponding author upon reasonable request.
