# Supplementary material for: Natural Sweetener Stevioside‐Based Dissolving Microneedles Solubilize Minoxidil for the Treatment of Androgenic Alopecia
Source: Adv Healthc Mater. 2025 Oct 7;15(2):e03575. doi: 10.1002/adhm.202503575 (PMC12805625; doi:10.1002/adhm.202503575)
Supplement: Supplementary file 1 — Supporting Information [file ADHM-15-0-s001.docx]

Natural sweetener stevioside-based dissolving microneedles solubilize minoxidil for the treatment of androgenic alopecia

Junying Zhang 1, $, Tianyu Shao 1, $, Hailiang Li 1, Luying Zhu 1, Lamyaa Albakr 2,3, Nial J. Wheate 4, Lifeng Kang 2, *, Chungyong Wu 5, *

^1^ Department of TCMs Pharmaceuticals, China Pharmaceutical University, Nanjing 210009, China

^2^ School of Pharmacy, Faculty of Medicine and Health, University of Sydney, Pharmacy and Bank Building A15, NSW 2006, Australia

^3^ Department of Pharmaceutics, College of Pharmacy, King Saud University, 11451 Riyadh, Saudi Arabia

^4^ School of Natural Sciences, Faculty of Science and Engineering, Macquarie University, NSW 2109, Australia

^5^ Department of Pharmaceutical Analysis, China Pharmaceutical University, Nanjing 210009, China

^$^ Junying Zhang and Tianyu Shao contributed equally to this work.

* Corresponding authors: Lifeng Kang, **lifeng.kang@sydney.edu.au**; Chunyong Wu, **cywu@cpu.edu.cn**

**Supporting Information (SI) 1.** Formula derivation

**Step 1: Start with the mass balance equations**

$\delta_{obs}=\delta_{mon}\frac{C_{mon}}{C_{t}}+\delta_{mic}\frac{C_{mic}}{C_{t}}$ (1)

Rearrange to get two essential relationships:

$\delta_{obs}-\delta_{mon}=\frac{C_{mic}}{C_{t}}(\delta_{mic}-\delta_{mon})$ (2)

${\delta_{mic}-\delta}_{obs}=\frac{C_{mon}}{C_{t}}(\delta_{mic}-\delta_{mon})$ (3)

**Step 2: Introduce the equilibrium constant** ^[1]^

$K=\frac{C_{mic}}{n{{(C}_{mon})}^{n}}$ (4)

$C_{mic}=nK{{(C}_{mon})}^{n}$ (5)

**Step 3: Combine and apply logarithms**

Divide equation (2) and (3) to eliminate the (δ_mic_​−δ_mon_​) term:

$\frac{\delta_{obs}-\delta_{mon}}{{\delta_{mic}-\delta}_{obs}}=\frac{C_{mic}}{C_{mon}}$ (6)

Substitute the expression for C_mic_​ from equation (5):

$\frac{\delta_{obs}-\delta_{mon}}{{\delta_{mic}-\delta}_{obs}}=nK{{(C}_{mon})}^{n-1}$ (7)

${log(\delta}_{obs}-\delta_{mon})- {{log(\delta}_{mic}-\delta}_{obs})=log(nK)+(n-1)logC_{mon}$ (8)

#### Step 4: Final rearrangement

From equation (3), solve for C_mon_​:

$C_{mon}=C_{t}\frac{{\delta_{mic}-\delta}_{obs}}{\delta_{mic}-\delta_{mon}}$ (9)

Substitute C_mon_ into the equation (8) and rearrange to arrive at the final form:

${log(\delta}_{obs}-\delta_{mon})- {{log(\delta}_{mic}-\delta}_{obs})=log(nK)+(n-1)log(C_{t}\frac{{\delta_{mic}-\delta}_{obs}}{\delta_{mic}-\delta_{mon}})$

(10)

${log(\delta}_{obs}-\delta_{mon})- {{log(\delta}_{mic}-\delta}_{obs})=log(nK)+(n-1)[logC_{t}+ log({\delta_{mic}-\delta}_{obs})-log(\delta_{mic}-\delta_{mon})]$ (11)

${log(\delta}_{obs}-\delta_{mon})- {{log(\delta}_{mic}-\delta}_{obs})=log(nK)+(n-1)logC_{t}+ (n-1)log({\delta_{mic}-\delta}_{obs})- (n-1)log(\delta_{mic}-\delta_{mon})$ (12)

$${log(\delta}_{obs}-\delta_{mon})=nlog({\delta_{mic}-\delta}_{obs})+log(nK)+nlogC_{t}-logC_{t}$$

$-\left( n-1 \right)log\left( \delta_{mic}-\delta_{mon} \right)$ (13)

$${log(\delta}_{obs}-\delta_{mon})+logC_{t}=nlogC_{t}+nlog({\delta_{mic}-\delta}_{obs})+log(nK)$$

$-\left( n-1 \right)log\left( \delta_{mic}-\delta_{mon} \right)$ (14) ${log[C_{t}(\delta}_{obs}-\delta_{mon})]=nlog[C_{t}({\delta_{mic}-\delta}_{obs})]+log(nK) +\left( 1-n \right)log\left( \delta_{mic}-\delta_{mon} \right)$ (15)

**SI 2.** Microneedle patches of STV tip solutions of different concentrations.


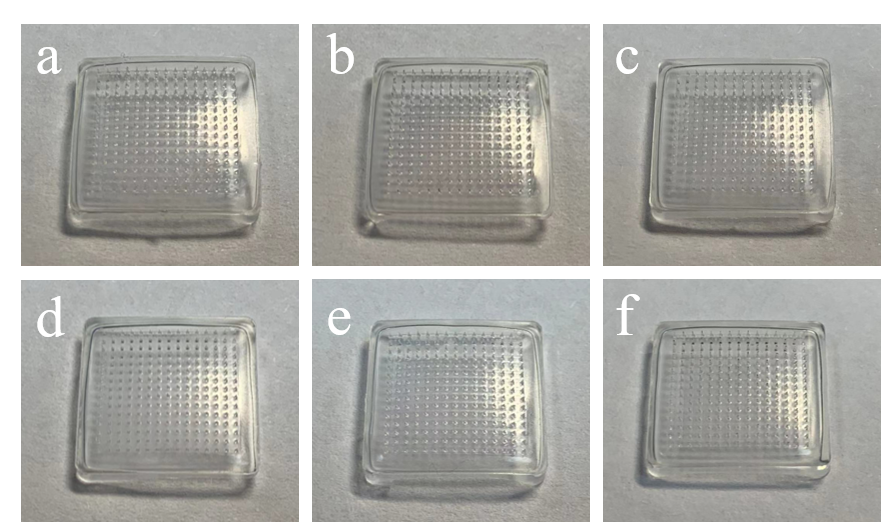


**Figure S1. Microneedle patches of STV tip solutions of different concentrations.** a.200 mg/mL; b.500 mg/mL; c.1000 mg/mL; d.1500 mg/mL; e.2000 mg/mL; f.3000 mg/mL.

**References**

[1] O. Söderman, P. Guering, *Colloid. Polym. Sci.* 1987, *265*, 76.
